# Non-pharmacological interventions to prevent PICS in critically ill adult patients: a protocol for a systematic review and network meta-analysis

**DOI:** 10.1186/s13643-024-02542-z

**Published:** 2024-05-14

**Authors:** Xiaoying Sun, Qian Tao, Qing Cui, Yaqiong Liu, Shouzhen Cheng

**Affiliations:** 1 School of Nursing, Sun Yat-Sen University, Guangzhou, 510080 China; 2 The First Affiliated Hospital of Sun Yat-Sen University, Guangzhou, 510080 China; 3 Department of Respiratory and Intensive Care Medicine, Shenzhen People’s Hospital, Shenzhen, 518020 China; 4 School of Nursing, Suzhou Medical College, Soochow University, Suzhou, 215006 China

**Keywords:** Postintensive care syndrome, Non-pharmacological intervention, Systematic review, Network meta-analysis

## Abstract

**Background:**

Postintensive care syndrome (PICS) is common in critically ill adults who were treated in the intensive care unit (ICU). Although comparative analyses between types of non-pharmacological measures and usual care to prevent PICS have been performed, it remains unclear which of these potential treatments is the most effective for prevention.

**Methods:**

To obtain the best evidence for non-pharmaceutical interventions in preventing PICS, a systematic review and Bayesian network meta-analyses (NMAs) will be conducted by searching nine electronic databases for randomized controlled trials (RCTs). Two reviewers will carefully screen the titles, abstracts, and full-text papers to identify and extract relevant data. Furthermore, the research team will meticulously check the bibliographic references of the selected studies and related reviews to discover any articles pertinent to this research. The primary focus of the study is to examine the prevalence and severity of PICS among critically ill patients admitted to the ICU. The additional outcomes encompass patient satisfaction and adverse effects related to the preventive intervention. The Cochrane Collaboration’s risk-of-bias assessment tool will be utilized to evaluate the risk of bias in the included RCTs. To assess the efficacy of various preventative measures, traditional pairwise meta-analysis and Bayesian NMA will be used. To gauge the confidence in the evidence supporting the results, we will utilize the Confidence in NMA tool.

**Discussion:**

There are multiple non-pharmacological interventions available for preventing the occurrence and development of PICS. However, most approaches have only been directly compared to standard care, lacking comprehensive evidence and clinical balance. Although the most effective care methods are still unknown, our research will provide valuable evidence for further non-pharmacological interventions and clinical practices aimed at preventing PICS. The research is expected to offer useful data to help healthcare workers and those creating guidelines decide on the most effective path of action for preventing PICS in adult ICU patients.

**Systematic review registration:**

PROSPERO CRD42023439343.

**Graphical Abstract:**

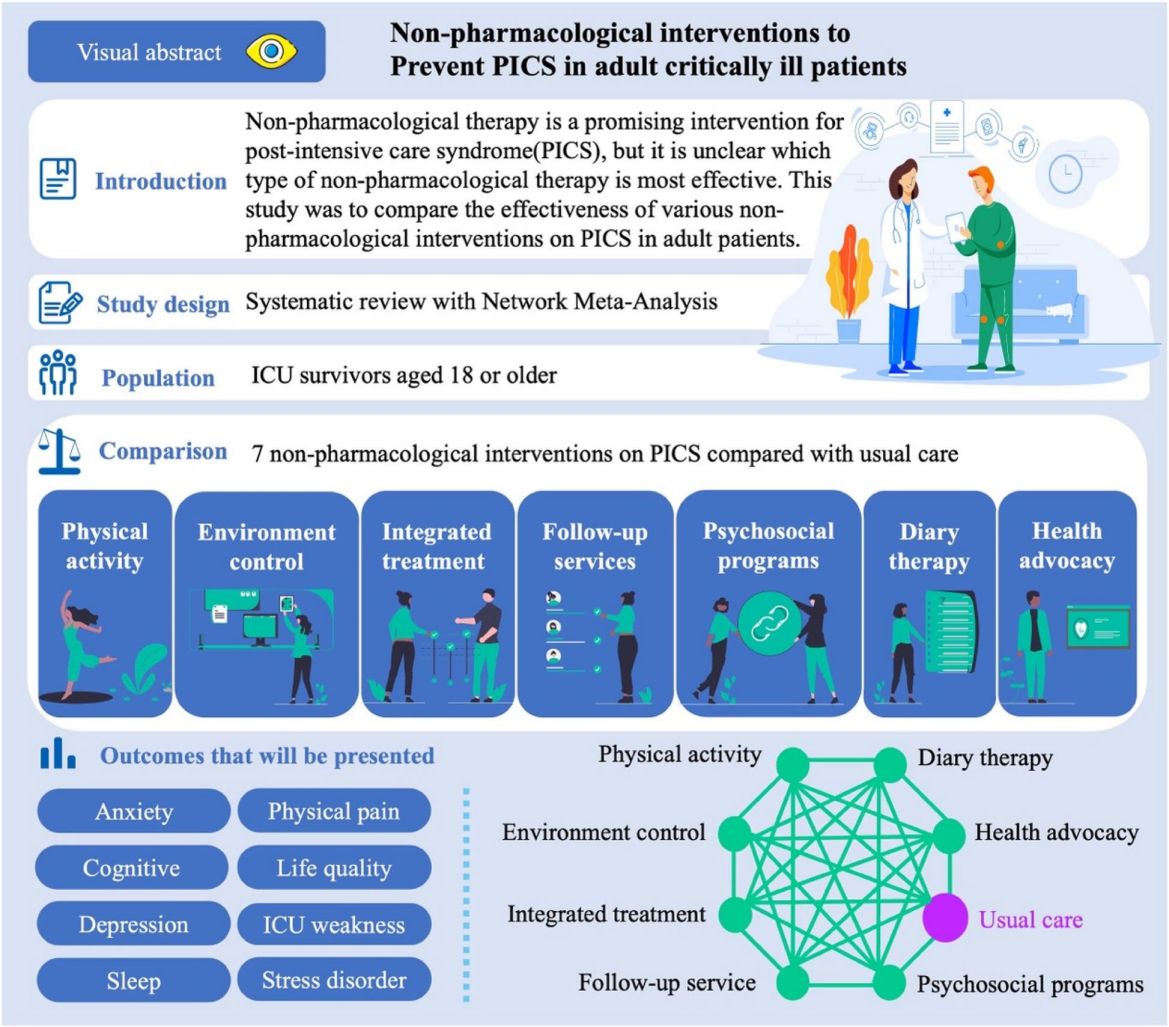

**Supplementary Information:**

The online version contains supplementary material available at 10.1186/s13643-024-02542-z.

## Background

Postintensive care syndrome (PICS) is an umbrella term used to define the general influence of severe disease on individuals who were treated in the intensive care unit (ICU), encompassing various physical (such as neuromuscular weakness and limitations in daily activities), psychological (such as anxiety, sadness, and post-traumatic stress disorder [PTSD]), and cognitive dysfunction [1–3]. These ailments impair everyday living and quality of life. A majority of adult patients who received treatment in the ICU encounter such impairments [4–6]. The significant progress made in the medical, scientific, and technological domains has led to a notable increase in survival among people admitted to the ICU in recent years [7]. However, although adults treated in the ICU have increased survival, their quality of life can be negatively affected by their time in the ICU.

**Fig. 1:**
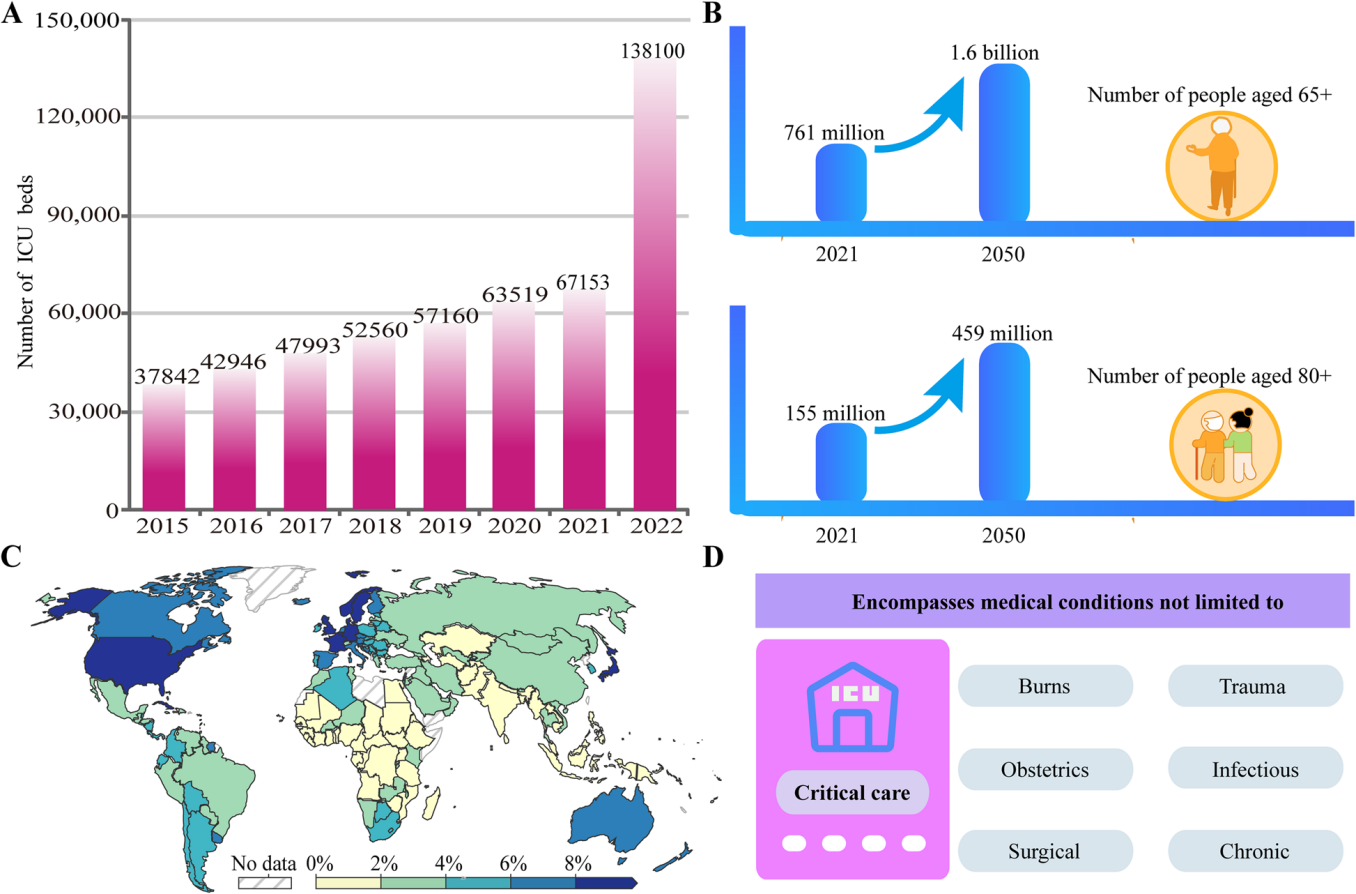
The burden of PICS is increasing. PICS, postintensive care syndrome

Intensive care is the medical care provided to critically ill patients during a medical emergency or crisis, managing severe conditions of all disease types [8]. Infectious and noninfectious illnesses and injuries contribute significantly to the global burden, with an increasing trend over the years. The Global Burden of Disease project does not provide specific information on the burden of critical illness and global variation [9–11]. Figure 1 describes the burden of critical illness based on global overall expenditure, the aging trend, and the number of ICU beds. These data come from Our World in Data [12], the China Health Statistics Yearbook [13], and United Nations Aging data [14].

In the past 50 years, the number of patients admitted to the ICU has continuously increased, especially after the beginning of the COVID-19 pandemic [15, 16]. This trend is evident from Fig. 1A, which shows the growth of ICU bed capacity in China. The percentage of public health spending as a part of the gross domestic product for each country in 2019 is shown in Fig. 1C. Developed countries invest more in the healthcare sector [17], which is likely closely related to their aging population and advancements in medical technology [18]. Figure 1B illustrates the projected future extent of global aging, indicating that the global population of individuals aged 65 years or older is expected to double within the next three decades, reaching an estimated 1.6 billion by 2050. Concurrently, the number of people aged 80 and older is anticipated to reach 459 million. The increase in age in the global population has led to a higher risk of critical illnesses, as the aging population bears a heavier load of chronic diseases [19]. However, the spectrum of medical conditions managed in the ICU includes not only the exacerbation of chronic diseases but also burns, trauma, and infectious diseases, as detailed in Fig. 1D. Moreover, our enhanced ability to treat formerly fatal conditions has led to higher demand for critical care services [20]. Consequently, PICS is also likely to increase with the growing number of adults treated in and discharged from the ICU.

Considering the substantial public health concerns arising from the consequences of PICS on quality of life, healthcare expenditures, and hospital readmissions, it is imperative to offer effective and feasible interventions to address this issue [21]. Assistance and support for patients in critical condition are potential interventions for improving outcomes related to PICS [22]. A recent study showed that administration of dexmedetomidine during the night as a preventive measure led to a substantial decrease in the incidence of PICS, as evidenced by a substantial reduction in psychological impairment during the 6-month monitoring period [23]. However, pharmacological treatments are often expensive and can pose a certain economic burden. Further, the use of sedative and anxiolytic drugs to treat patient symptoms is linked to delirium and negative physical and mental health consequences [24]. Consequently, there is an increasing focus on employing non-pharmacological approaches and establishing a more person-centered atmosphere within the ICU, aiming to benefit both patients and their families [25].

The current interventions for PICS that show the most potential involve non-pharmacological strategies [22]. The efficacy of early rehabilitation treatment, which consists of all physiotherapy, occupational therapy, and palliative care-related support, in managing PICS was explored through a systematic review [7], which showed that such treatment can lead to an improvement in short-term physical functioning but does not have any impact on mental or cognitive aspects. ICU diaries can reduce ICU-related psychological complications, such as ICU-related PTSD, depression, and anxiety [26]. However, results obtained from a randomized controlled trial (RCT) indicate that the use of ICU diaries alone does not provide any advantage over bedside education in reducing the symptoms of PTSD that are related to the stay in ICU [27]. Hence, it is still uncertain which non-pharmacological interventions are the most effective and preferred in preventing depression and anxiety, cognitive disorder, and physical function for adults with critical illness.

Despite the potential deleterious effects of PICS in terms of healthcare usage and caregiver burden and the increasing population of adults treated in and discharged from ICU, there is a lack of evidence-based practices for this specific group [28]. Although they provide indirect evidence to evaluate the confidence of treatment comparisons, network meta-analyses (NMAs) [29] have substantial advantages over conventional pairwise meta-analyses. NMAs allow for the evaluation of comparative effects that have not been directly compared in RCTs, potentially yielding more reliable and conclusive outcomes [30]. Hence, the study’s main goal is to use NMA to examine several non-pharmacological preventative treatments that addressed PICS in individuals treated in the ICU.

## Methods/design

### Criteria for eligibility

#### Setting

Studies conducted during the ICU stay, as well as those extending from the ICU admission through to the post-discharge period, will be eligible for inclusion.

#### Participants

Adults (aged > 18 years) admitted to the ICU were included in the study. Gender, ethnicity, and nationality of participants will not be further restricted.

#### Type of study

Only RCTs providing comparisons of preventative strategies and other strategies or standard treatment for adult patients in ICUs with full-text publications will be included.

#### Intervention

Any non-pharmacological interventions to prevent PICS in critically ill patients. The potential interventions may encompass, but are not limited to the following:

- Psychosocial programs
- Follow-up service
- Patient instructions
- Exercise (e.g., strength and cardiovascular exercise)
- Diary therapy
- Environment control
- Integrated therapy

#### Comparators

These are different types of non-pharmacological interventions or a control group; a control group is defined as a waiting list, usual/standard care, or a control condition that provided a brief educational leaflet.

#### Outcome measures

Studies must have assessed depression symptoms, anxiety symptoms, PTSD, cognitive status, sleep quality, pain, physical functioning, or quality of life, with detailed data available. Additionally, the evaluation of primary outcomes must use a comprehensive and specific scale, including but not limited to the following:

##### Primary outcomes

1. Depression: Hospital Anxiety and Depression Scales [31] and Hamilton Depression Rating Scale [32]
2. Anxiety: Hospital Anxiety and Depression Scales [31]
3. PTSD: The Impact of Event Scale-Revised [33] and the Davidson Trauma Scale [34]
4. Cognitive: The Confusion Assessment Method for the ICU [35] and Montreal Cognitive Assessment [36]
5. Sleep: Richards Campbell Sleep Questionnaire [37] and Pittsburgh Sleep Quality Index [38]
6. Pain: Numeric rating scale [39] and visual analog scale [40]
7. Physical functioning: The occurrence rate of ICU-acquired weakness and the evaluation through Medical Research Council scale scores [41] and activities of daily living [42, 43]
8. Quality of life: Medical Outcomes Study 36-item short-form health survey [44] and European Quality of Life-5 Dimensions questionnaire [45]

##### Secondary outcomes

1. Any harms associated with the prevention intervention
2. Participant satisfaction

### Search strategy

“Critical care,” “intensive care units,” “syndrome,” “symptom assessment,” “depression symptom,” “depression,” “anxiety,” “anxiety symptom,” “mental health,” “Posttraumatic Stress Disorder,” “cognitive dysfunction,” “delirium,” “sleep,” “sleep wake disorder,””sleep quality,””pain,””intensive care unit acquired weakness,” and “physical functioning” will be utilized as MeSH phrases or keywords. The following electronic databases will be search from inception to June 25, 2023: PubMed, Embase, CINAHL, Cochrane Central Register of Controlled Trials, Web of Science, PsycINFO, SinoMED, CNKI, and Wangfang. Example searches of PubMed can be found in Table 1. Moreover, we will perform thorough reverse citation searches on all included studies and pertinent reviews to find any previously missed references. Additionally, to find recent articles that have mentioned the pertinent literature, we will do forward reference searching on Google Scholar. Finally, we will try to contact the authors of those studies for more information if the full text of certain sources is unavailable.

**Table 1:** Search strategy in PubMed

Order	Search items
#1	MeSH terms: “postintensive care syndrome”
#2	Title/abstract: “PICS” OR “post-intensive care syndrome” OR “post ICU syndrome”
#3	#1OR#2
#4	MeSH terms: “Aftercare” OR “Counseling” OR “Exercise Therapy” OR “Occupational Therapy” OR “Physical Therapy Modalities” OR “Psychotherapy” OR “Rehabilitation” OR “Music Therapy” OR “Mindfulness” OR “Cognitive Behavioral Therapy”
#5	Title/abstract: “nonpharmacol*” OR “non-pharmacol*” OR “anxiety management” OR “consultation*” OR “counselling” OR “diaries” OR “diary” OR “early exercise” OR “early mobilisation” OR “intervention*” OR “mobilit*” OR “physical therap*” OR “psychoeducation” OR “psycho-education” OR “psychosocial support group” OR “program*” OR “psychotherap*” OR “therap*” OR “training” OR “CBT” OR “cognitive therapy”
#6	#4 OR #5
#7	MeSH terms: “Critical Care Nursing” OR “Critical Care” OR “Critical Illness” OR “Intensive Care Units”
#8	MeSH terms: “Fatigue” OR “Mobility limitation” OR “Muscle Weakness” OR “Pain” OR “Dyssomnias” OR “Anxiety” OR “Depression” OR “Stress Disorders, Post-Traumatic”
#9	Title/abstract: “physical decline” OR “physical disability” OR “ICUAW” OR “ICU acquired weakness” OR “sleep disorder” OR “PTSD” OR “mental disorder” OR “mental symptoms” OR “cognitive impairment” OR “Cognitive dysfunction”
#10	#8 OR #9
#11	Publication type: “Randomized Controlled Trial”
#12	MeSH terms: “Randomized Controlled Trials as Topic”
#13	Title/abstract: “randomized” OR “randomly” OR “RCT” OR “trial”
#14	#11 OR #12 OR #13
#15	#3 AND #6 AND #14
#16	#6 AND #7AND #10 AND #14
#17	#15 OR #16

*MeSH* Medical Subject Headings

### Study selection

**Fig. 2:**
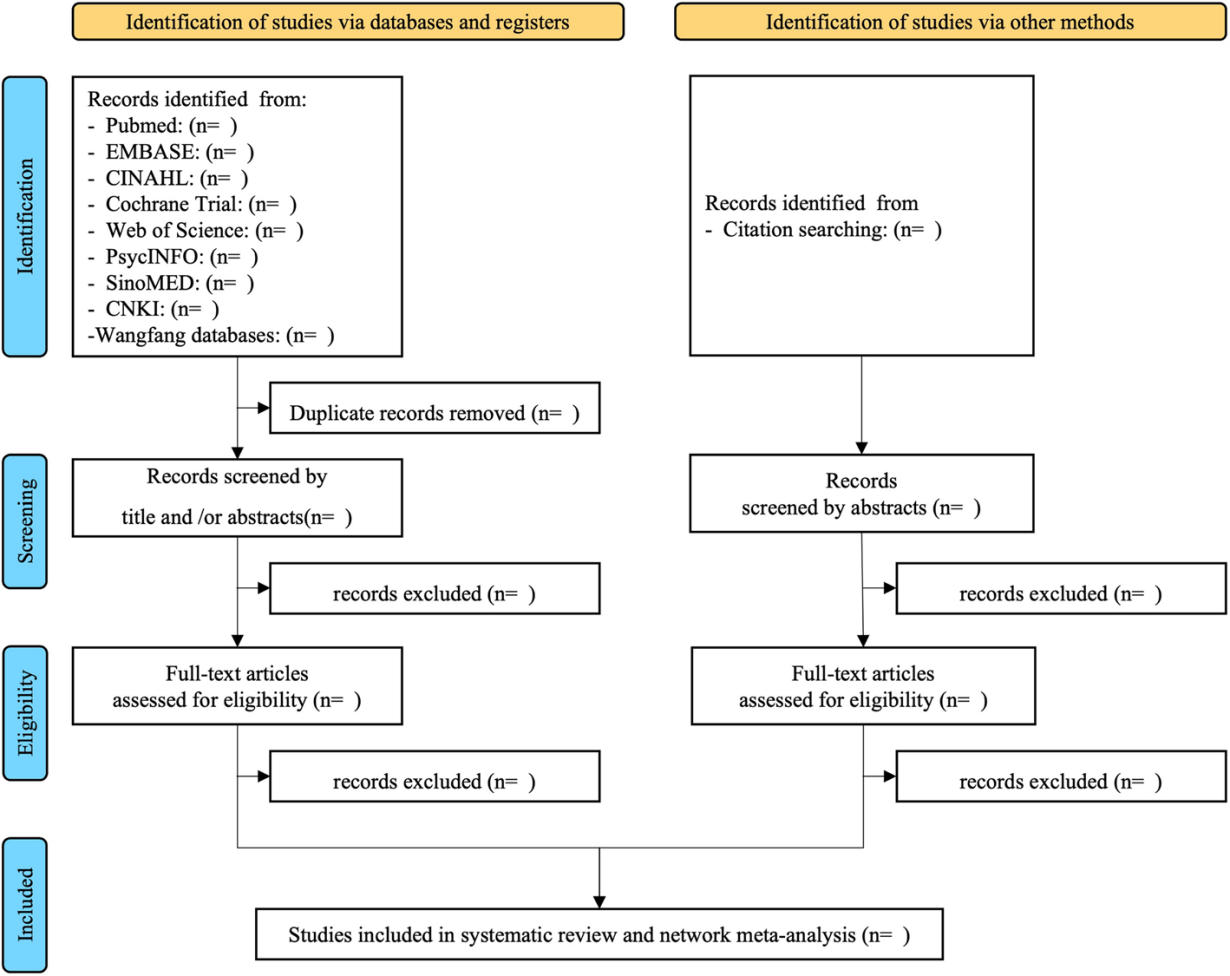
Study selection

This study will follow the Preferred Reporting Items for Systematic Reviews and Meta-Analyses criteria, and the Preferred Reporting Items for Systematic Reviews and Meta-Analyses flow diagram [46], shown in Fig. 2, demonstrates the proposed research selection methods. The discovered studies will be imported into the online Rayyan literature management tool (https://rayyan.qcri.org) for additional analysis. Independent screening of the papers’ titles and abstracts will be performed by two reviewers. If either reviewer determines that an article meets the inclusion criteria, full texts will be obtained. Subsequently, both reviewers will independently assess the eligibility of each reference through a thorough examination of the full text. Any differences that cannot be settled via conversation will be brought to the attention of a third reviewer who will act as a mediator. Cohen’s kappa coefficient will be calculated to measure the inter-rater reliability. The reasons for excluding any studies will be carefully documented.

### Data extraction

A standardized data extraction form is available as a supplemental file. Before the actual usage of the form, each member of the team will have the opportunity to test it. Two reviewers will independently perform data extraction. In the case of any inconsistencies, a third arbiter will be consulted to facilitate a discussion and achieve a consensus. Our inclusion criteria for data extraction include various aspects of the study, such as background data (first contributor and the time of publication), research design (setting, methods of sampling, randomization, allocations, and blinding), sample characteristics (inclusion and exclusion criteria, sample size, age, sex, and educational background, rates, or severity of PICS), intervention details (type, content, frequency, duration, provider, and control group), and primary and secondary outcomes (including measurement time points, assessment tools, and any negative effects connected to preventative measures). In cases where information is missing or requires further clarification, we will reach out to the corresponding author for additional details.

### Risk of bias

Two individuals will independently determine the risk of bias. If a dispute or discrepancy cannot be settled via conversation, a third reviewer will help achieve an agreement. We will weigh the RCTs’ quality of methodology using the revised Cochrane risk-of-bias methodology for randomized trials [47]. The five domains of this tool are as follows: (1) risk of bias resulting from the randomization process, (2) risk of bias due to departure from the purpose of the intervention, (3) risk of bias due to lacking outcome data, (4) risk of bias in measuring of the outcome, and (5) risk of bias in selection of the presented result.

### Data synthesis

Study results will be categorized and summarized based on the intervention type, detailing the methodologies and clinical attributes documented in the corresponding studies. The summary will include an exhaustive analysis of patient demographics, the reported outcomes, and a critical assessment of potential bias risks. In instances where a quantitative synthesis of research findings is infeasible, a narrative synthesis will elucidate the systematic reviews outcomes.

### Assessment of transitivity

In NMA, the transitivity assumption is crucial, allowing for indirect comparisons between interventions via a common comparator [48]. Considering the inherent clinical and methodological diversity in systematic reviews, it is essential for researchers to determine whether such variability could significantly impact the transitivity. To identify potential intransitivity, we will scrutinize the distribution of known effect modifiers across all direct comparisons before conducting the NMA [49], including variables like age, gender, disease severity, and the duration of interventions. A comparable distribution of these factors suggests that the transitivity assumption holds. Conversely, if transitivity is compromised, the NMA results may be biased, warranting a more conservative interpretation.

### Network meta-analysis

Should the assumption of transitivity be deemed met, a random-effects NMA [50] will be executed employing vague priors within a Bayesian framework.

### Detection of heterogeneity

Considering the anticipated variability in participant demographics, intervention methodologies, and outcome measurements, statistical heterogeneity is expected. In anticipation of inherent variability across the included studies, we will implement a random-effects model to mitigate the observed statistical heterogeneity. The deviance information criterion (DIC) will serve as our comparative metric for model selection, integrating considerations of model fit with complexity.

To explore the sources of heterogeneity, we will conduct network meta-regression, subgroup analyses, and sensitivity analyses [51]. Network meta-regression will be carried out to examine the impact of potential effect modifiers (e.g., average age of participants, baseline symptom scores) on the primary outcomes. The duration of interventions may be a significant factor affecting efficacy, and subgroup analyses will be performed to assess the influence of different intervention durations on the primary outcomes. Additionally, if a sufficient number of studies are available, we will conduct sensitivity analyses by excluding trials assessed to be at high risk of bias to ensure the robustness of the primary study results.

### Assessment of inconsistency

When closed loops are present within the NMA framework, the node-splitting approach is employed to evaluate the consistency between direct and indirect evidence. *p* > 0.05 in the node-splitting analysis is indicative of agreement between the two sources [52].

### Assessment of publication bias

In instances where a treatment comparison encompasses over 10 studies, we will utilize a comparison-adjusted funnel plot to evaluate potential small-study effects and the likelihood of publication bias [53]. The symmetry of these plots will be systematically assessed via Egger’s test.

The overall strength of the evidence will be assessed while accounting for research limitations, imprecision, heterogeneity, indirectness, and publication bias using the Confidence in Network Meta-Analysis (CINeMA) method. The Grading of Recommendations Assessment, Development and Evaluation (GRADE) framework is the foundation of CINeMA [54] and contains the following six dimensions: within-study bias, reporting bias, indirectness, imprecision, heterogeneity, and incoherence. The adoption of CINeMA boosts transparency and prevents the selective use of evidence in making judgments, thereby reducing the level of subjectivity.

### Statistical analyses

All studies will be performed using the R-evolution software [55] version 4.3.0 and the gemtc package [56] version 1.0–1, which connects with JAGS version [57] 4.3.2 to perform a Markov chain Monte Carlo simulation (MCMC) [58]. We will configure 4 Markov chains, with executing a minimum of 20,000 iterations. The concordance between direct and indirect evidence will be ascertained through the node-splitting technique. Model convergence will be gauged using convergence diagnostic and trace density plots, with the potential scale reduction factor (PSRF) providing a metric for convergence adequacy—a PSRF close to 1 suggests satisfactory convergence. For continuous outcomes, the mean difference (MD) is utilized as the measure of effect, whereas for binary outcomes, the risk ratio (RR) is used, including its 95% confidence interval (CI). The area under the cumulative ranking curve (SUCRA), as determined from the ranking probability matrix generated by R software, will be calculated and the corresponding SUCRA curve plotted; a greater SUCRA value indicates an increased likelihood of a superior outcome ranking.

A network diagram will be created to visualize relationships between interventions [59]. Data processing will be executed utilizing network group commands. Subsequent to this, network evidence graphs will be generated [58]. In these visual representations, the magnitude of the nodes will be proportional to the sample sizes derived from the comparative analysis of interventions. The thickness of the edges will represent the volume of RCTs interlinking the interventions.

## Discussion

The ICU is a specialized hospital department dedicated to the intensive care and treatment of seriously ill patients. The recovery of patients treated in the ICU is crucial for their well-being, as well as for their families and society [60]. However, ICU patients experience a decline in immunological response and hormone disruption owing to the nature of their illnesses and the risk factors during ICU treatment [61]. This can lead to various symptoms, including sleep disturbance, anxiety, depression, cognitive impairment, and PTSD. Individuals can exhibit one or multiple symptoms of PICS [21], and they significantly impact the patient’s quality of life and impose additional economic and caregiving burdens on society. Current preventive measures for PICS in ICU patients mainly comprise pharmacological and non-pharmacological interventions. Non-pharmacological interventions primarily involve physical activity, ICU diaries, psychotherapy, health education, and comprehensive treatment [62, 63]. However, there is no research evaluating the most effective non-pharmacological preventive measures. Therefore, this proposed study aims to compare the occurrence of PICS using an NMA approach to assess the effectiveness of various intervention measures.

The proposed systematic review and NMA aim to address the effectiveness of intervention measures in preventing PICS in adults treated in the ICU. Developing effective preventive interventions can help alleviate the social and economic burden of PICS by reducing new cases or alleviating symptoms in affected individuals. This systematic review will employ NMA to compare all non-pharmacological measures aimed at preventing PICS. The primary outcomes will include the incidence or relief of various PICS symptoms, such as depression, anxiety, PTSD, cognitive impairment, sleep disturbance, physical functional impairment, and pain. Secondary outcomes include participant satisfaction and the frequency of adverse events.

To the best of our knowledge, this will be the first systematic review and NMA to evaluate currently available non-pharmacological therapies for preventing PICS. The research findings will provide rankings in terms of treatment effectiveness and acceptability, which will contribute to evidence-based decision-making in the rehabilitation of ICU patients and further development of other non-pharmacological interventions. Furthermore, the methodology of this protocol is based on the Cochrane Handbook for Intervention Reviews [64], the PRISMA statement [46, 65], and GRADE assessment [66], taking into account the risks of random errors and systematic errors.

The ability of our systematic review and NMA to draw conclusions about non-pharmacological interventions for PICS in individuals treated in the ICU may be limited by the available data, which could be considered a limitation of this study. However, despite this limitation, identifying the best available evidence from current research is still valuable. Additionally, we will search only Chinese and English databases and will not analyze articles in other languages, which may be another limitation. However, it is worth noting that the majority of high-quality studies are usually published in English and included in English databases, so our analysis is unlikely to omit important studies.

Some current trials may not have included patient preferences [67], but our study originates from previous research and uses existing outcome data for statistical analysis. Therefore, we hope individuals treated in the ICU can make their own choices combined with their circumstances while receiving prevention recommendations from doctors based on clinical evidence.

### Supplementary Information

**Additional file 1.** PRISMA-P 2015 Checklist. Data extraction form

## Data Availability

The study is a systematic review.

## Abbreviations

CINeMA: Confidence in Network Meta-Analysis
GRADE: Grading of Recommendations Assessment, Development, and Evaluation
ICU: Iintensive care unit
NMA: Network meta-analysis
PICS: Postintensive care syndrome
PTSD: Posttraumatic stress disorder
RCT: Randomized controlled trial
PRISMA: Preferred Reporting Items for Systematic Reviews and Meta-Analyses
DIC: The deviance information criterion
MCMC: Markov chain Monte Carlo simulation
PSRF: Potential scale reduction factor
MD: Mean difference
RR: Risk ratio
CI: Confidence interval
SUCRA: Area under the cumulative ranking curve
