# Supplementary material for: Non-pharmacological interventions to prevent PICS in critically ill adult patients: a protocol for a systematic review and network meta-analysis
Source: Syst Rev. 2024 May 14;13:132. doi: 10.1186/s13643-024-02542-z (PMC11095022; doi:10.1186/s13643-024-02542-z)
Supplement: Supplementary file 1 — Additional file 1. PRISMA-P 2015 Checklist. Data extraction form [file 13643_2024_2542_MOESM1_ESM.docx]

**Non-pharmacological interventions to prevent PICS in adult critically ill patients: a protocol for a systematic review and network meta-analysis**

[PRISMA-P 2015 Checklist 2](#_Toc140649614)

[Data extraction form 6](#_Toc140649615)

# PRISMA-P 2015 Checklist

This checklist from Table 3 in Moher D et al: Preferred reporting items for systematic review and meta-analysis protocols (PRISMA-P) 2015 statement. Systematic Reviews 2015 4:1

| **Section/topic** | **Item #** | **Checklist item** | **Information reported** | | |
| --- | --- | --- | --- | --- | --- |
|  |  |  | **Yes** | **NO** | **Not applicable** |
| **ADMINISTRATIVE INFORMATION** | | | | | |
| **Title** | | | | | |
| Identification | 1a | Identify the report as a protocol of a systematic review | ü |  |  |
| Update | 1b | If the protocol is for an update of a previous systematic review, identify as such |  |  | ü |
| Registration | 2 | If registered, provide the name of the registry (e.g., PROSPERO) and registration number in the  Abstract | ü |  |  |
| Authors | | | | | |
| Contact | 3a | Provide name, institutional affiliation, and e-mail address of all protocol authors; provide physical  mailing address of corresponding author | ü |  |  |
| Contributions | 3b | Describe contributions of protocol authors and identify the guarantor of the review | ü |  |  |
| Amendments | 4 | If the protocol represents an amendment of a previously completed or published protocol, identify  as such and list changes; otherwise, state plan for documenting important protocol amendments |  |  | ü |
| Support | | | | | |
| Sources | 5a | Indicate sources of financial or other support for the review |  |  | ü |
| Sponsor | 5b | Provide name for the review funder and/or sponsor |  |  | ü |
| Role of  sponsor/funder | 5c | Describe roles of funder(s), sponsor(s), and/or institution(s), if any, in developing the protocol |  |  | ü |
| INTRODUCTION | | | | | |
| Rationale | 6 | Describe the rationale for the review in the context of what is already known | ü |  |  |
| Objectives | 7 | Provide an explicit statement of the question(s) the review will address with reference to  participants, interventions, comparators, and outcomes (PICO) | ü |  |  |
| Eligibility criteria | 8 | Specify the study characteristics (e.g., PICO, study design, setting time frame) and report  characteristics (e.g., years considered, language, publication status) to be used as criteria for  eligibility for the review | ü |  |  |
| Information sources | 9 | Describe all intended information sources (e.g., electronic databases, contact with study authors,  trial registers, or other grey literature sources) with planned dates of coverage | ü |  |  |
| Search strategy | 10 | Present draft of search strategy to be used for at least one electronic database, including planned  limits, such that it could be repeated | ü |  |  |
| STUDY RECORDS | | | | | |
| Data management | 11a | Describe the mechanism(s) that will be used to manage records and data throughout the review | ü |  |  |
| Selection process | 11b | State the process that will be used for selecting studies (e.g., two independent reviewers) through  each phase of the review (i.e., screening, eligibility, and inclusion in meta-analysis) | ü |  |  |
| Data collection  process | 11c | Describe planned method of extracting data from reports (e.g., piloting forms, done independently,  in duplicate), any processes for obtaining and confirming data from investigators | ü |  |  |
| Data items | 12 | List and define all variables for which data will be sought (e.g., PICO items, funding sources), any  pre-planned data assumptions and simplifications | ü |  |  |
| Outcomes and  prioritization | 13 | List and define all outcomes for which data will be sought, including prioritization of main and  additional outcomes, with rationale | ü |  |  |
| Risk of bias in  individual studies | 14 | Describe anticipated methods for assessing risk of bias of individual studies, including whether this  will be done at the outcome or study level, or both; state how this information will be used in data  synthesis | ü |  |  |
| DATA | | | | | |
| Synthesis | 15a | Describe criteria under which study data will be quantitatively synthesized | ü |  |  |
|  | 15b | If data are appropriate for quantitative synthesis, describe planned summary measures, methods of  handling data, and methods of combining data from studies, including any planned exploration of  consistency (e.g., /^2^, Kendall's tau) | ü |  |  |
|  | 15c | Describe any proposed additional analyses (e.g., sensitivity or subgroup analyses, meta-  regression) | ü |  |  |
|  | 15d | If quantitative synthesis is not appropriate, describe the type of summary planned | ü |  |  |
| Meta-bias(es) | 16 | Specify any planned assessment of meta-bias(es) (e.g., publication bias across studies, selective  reporting within studies) | ü |  |  |
| Confidence in  cumulative evidence | 17 | Describe how the strength of the body of evidence will be assessed (e.g., GRADE) | ü |  |  |

# Data extraction form

| Subject | Content |
| --- | --- |
| Publication information | First author and correspondence author, e-mail, publish year, country, corporate sponsorship |
| Participant | Recruitment source, sample size, age, gender, diagnostic criteria, type of PICS, inclusion and exclusion criteria |
| Intervention | Non-pharmacological therapy name, specific intervention measures, frequency of the therapy and the therapy course |
| Control | Usual care name, specific intervention measures, administration, dosage and usage, frequency of the therapy and the therapy course |
| Outcome | Outcome measurements and each assessment time point, measurement instruments, adverse events, and the detailed data |
| Study design | study sites, randomization and blinding, statistical analysis, sample size calculation |
| Other information | withdrawing |
